# Assessing fidelity to treatment delivery in the ICONS (Identifying Continence OptioNs after Stroke) cluster randomised feasibility trial

**DOI:** 10.1186/s12874-015-0051-9

**Published:** 2015-08-21

**Authors:** Brigit M. Chesworth, Michael J. Leathley, Lois H. Thomas, Christopher J. Sutton, Denise Forshaw, Caroline L. Watkins, The ICONS project team and the ICONS Patient, Public and Carer Involvement Groups

**Affiliations:** Clinical Practice Research Unit, School of Health, University of Central Lancashire, Preston, UK; Lancashire Clinical Trials Unit, School of Health, University of Central Lancashire, Preston, UK; Faculty of Health Sciences, Australian Catholic University, Sydney, Australia

**Keywords:** Intervention fidelity, Treatment fidelity, Treatment delivery, Stroke, Urinary incontinence, Systematic voiding programme

## Abstract

**Background:**

The implementation of strategies to monitor and enhance treatment fidelity is of paramount importance in trials of complex interventions. A recent framework published by the *National Institutes of Health Behavior Change Consortium* recommends addressing five areas of treatment fidelity, one of which is delivery of treatment. This study aimed to explore fidelity to treatment delivery of the ICONS intervention (a systematic voiding programme [SVP]). This included exploring the feasibility of a method to assess fidelity to treatment delivery and collecting preliminary evidence of the level of fidelity to SVP delivery in order to inform strategies for improving fidelity in a future trial.

**Methods:**

Delivery of treatment was recorded by nurses through completion of daily clinical logs, which included: a voiding interval, proposed voiding times and actual voiding times. The *a priori* method for assessment of fidelity – comparing actual voiding times with proposed voiding times – was trialled on a small amount of data. Due to errors in documentation of the voiding intervals and proposed voiding times it was not possible to assess fidelity directly as planned. A new method was devised, which included identification of ‘key quality indicators’.

**Results:**

This new approach to assessing fidelity used key quality indicators based upon presence of the data needed to make the comparison between proposed and actual voiding times. The proportion of clinical logs with correct documentation of voiding intervals and proposed voiding times was less than 40 %. For clinical logs with correct documentation, an actual voiding time within 30 min of the proposed voiding time was identified on approximately 55 % of occasions.

**Conclusions:**

Lessons learnt from this study have implications for the future ICONS definitive trial and for other trials of complex interventions. Implementation of a complex intervention may often deviate from what is intended. While careful consideration should be given to the best method of fidelity assessment, an iterative approach allowing flexibility to adapt pre-planned methods is recommended within feasibility trials. As fidelity to treatment delivery in the ICONS feasibility trial appeared to be relatively low, more attention to implementation strategies will be required in the definitive trial.

**Trial registration:**

Identifier: ISRCTN08609907; date registered: 07/07/2010.

**Electronic supplementary material:**

The online version of this article (doi:10.1186/s12874-015-0051-9) contains supplementary material, which is available to authorized users.

## Background

Treatment fidelity is of particular relevance to trials of complex interventions, because intervention delivery has the potential to differ substantially across research sites as well as over time. Treatment fidelity is defined as “…the methodological strategies used to monitor and enhance the reliability and validity of behavioral interventions…[and]…the methodological practices used to ensure that a research study reliably and validly tests a clinical intervention” [1]. The development of effective strategies to monitor and enhance treatment fidelity is of paramount importance in complex intervention trials for multiple reasons.

Measuring fidelity improves the internal validity of a study: information on fidelity to the intervention can help estimate the degree to which trial outcomes are attributable to the intervention itself, rather than to other, unknown factors [1–7]. Without measuring fidelity, researchers do not know whether non-significant outcomes reflect a lack of intervention effectiveness or a lack of intervention fidelity [1–3, 6].

Providing information about fidelity to intervention delivery can enhance a trial’s reproducibility and therefore increase its external validity [1, 2, 4, 7–9], as well as aiding successful dissemination and implementation of the intervention within clinical practice [1, 5, 6, 10]. Additionally, if fidelity is optimised and therefore variability in intervention delivery is minimised, then the statistical power of a study can be increased [1–3]. Fidelity monitoring also provides an understanding of “how and why an intervention works” [5], including whether certain ‘doses’ of an intervention are required for a positive outcome [2].

In 2004 the National Institutes of Health (NIH) Behavior Change Consortium (BCC) published recommendations on the implementation of treatment fidelity practices within health behaviour intervention research [1]. These recommendations include five areas of treatment fidelity: study design, provider training, treatment delivery, treatment receipt, and enactment of treatment skills [1]. Since the publication of these treatment fidelity recommendations, a number of studies of complex interventions have used the recommendations as a framework against which to address treatment fidelity [2, 3, 11–14], and the recommendations have been found to be a useful model [3].

There are various challenges to monitoring and enhancing treatment fidelity in complex intervention trials. Challenges may include constraints on the time, money and resources required in order to implement strategies for monitoring fidelity; variation in practice between different professionals delivering the intervention, and the characteristics of the local setting/population [3, 13, 15]. There may also be a conflict between the desire of researchers for strict compliance with the intervention protocol and the desire of intervention providers to adapt the intervention according to the local setting [15].

In this paper we describe how fidelity to ‘treatment delivery’, one of the five areas of the BCC framework [1], was assessed in the ‘Identifying Continence OptioNs after Stroke’ (ICONS) trial [16, 17]. This included exploring the feasibility of a method to assess fidelity and collecting preliminary evidence of the level of fidelity in order to inform strategies for improving fidelity in a future trial. We illustrate some of the challenges encountered in assessing fidelity to treatment delivery using the example of conservative interventions for urinary incontinence.

ICONS is a cluster randomised controlled feasibility trial designed to provide preliminary evidence of the effectiveness and cost-effectiveness of a systematic voiding programme (SVP) for the management of continence after stroke. Urinary incontinence (UI) is a common problem for patients following a stroke, affecting around half of patients in the acute phase [18–20]. Conservative interventions (such as bladder training and pelvic floor exercises) are recommended as the first-line management option for UI post-stroke [21]. The SVP specifies an algorithm-driven choice of conservative continence management strategies tailored to the type of urinary problem and the cognitive ability of the patient. Strategies include bladder training and prompted voiding.

Twelve stroke services in England and Wales were randomised to receive Usual Care (UC, *n* = 4), the SVP (‘Intervention’ sites, *n* = 4), or the SVP plus Supported Implementation (‘Supported Implementation’ sites, *n* = 4): consequently, eight sites in total delivered the SVP. The Intervention sites included a total of 164 patients and the Supported Implementation sites included a total of 125 patients. The Supported Implementation arm used facilitation, an implementation strategy involving supporting and enabling people to change their practice [22], to enhance embedding of the SVP.

### Aim

To explore fidelity to treatment delivery in the ICONS trial.

### Objectives

1. To explore the feasibility of a method to assess fidelity to treatment delivery.
2. To collect preliminary evidence of the level of fidelity to treatment delivery.
3. To identify strategies to enhance fidelity in a future trial.

## Methods

Within the ICONS trial there was a process evaluation, which included assessment of fidelity through an examination of: a) completion of intervention documentation (three day diaries and daily clinical logs for participants on bladder training and prompted voiding) and b) adherence to the protocol in terms of allocation of participants to the appropriate regime and the management of catheterisation.

This paper focuses on one aspect of fidelity – daily clinical logs. Other aspects of fidelity were also explored in the trial, and these findings are published in the full trial report [17]. However, this paper focuses on clinical logs because these constitute the SVP itself, which was felt to be the ‘core component’ [23] of the intervention. The clinical logs were used by healthcare staff, predominantly nursing staff (registered) and healthcare assistants (non-registered), to undertake and record delivery of the SVP each day. One clinical log was completed for each patient for each day that they received the SVP. There were two types of clinical logs corresponding to the two SVP regimes, prompted voiding and bladder training. The patient’s details and an appropriate voiding interval were recorded on the clinical log at the start of each day. The ‘voiding interval’ refers to the specified length of time between each void, e.g. “3-hourly”, and healthcare staff were asked to select an appropriate voiding interval for each patient at the beginning of every day. Based upon the voiding interval, healthcare staff were asked to record a schedule of proposed voiding times for the patient to follow throughout the day (from 7.30 am until 9.30 pm). Subsequently, any healthcare staff caring for the patient that day, which may have included the staff member who originally wrote the schedule, attempted to follow the schedule of proposed voiding times: the aim being for the patient to void within 30 min of each proposed voiding time. Thirty minutes’ leeway was agreed as a balance between expert opinion that voiding should occur within 10 min of the proposed voiding time and the need to be pragmatic and realistic when working in an acute or rehabilitation setting. After every void, healthcare staff were asked to document the actual voiding time and document whether a number of ‘best practice’ components had been achieved (e.g. giving encouragement; asking the patient if they were wet).

## Results

Healthcare staff were provided with written information in the form of an intervention protocol regarding how to undertake the SVP, and training was provided in the form of both online training and/or face-to-face training. However, uptake was sub-optimal. Details of training provision and uptake are provided in the full ICONS report [17].

In order to assess fidelity to treatment delivery of the SVP, daily clinical logs were sampled from all eight sites delivering the SVP. For each site, time periods of 14 days each were selected by the trial statistician using stratified sampling. The planned recruitment period for each site was split into three strata and two time periods of 14 days each were sampled from within each stratum. Sites which extended recruitment had an additional stratum added, with further 2-week periods sampled according to the duration of extension. This resulted in site samples comprising between six and nine 14-day periods. The sample of daily clinical logs comprised all logs for all participants on the SVP during the sampled 14-day periods. All available logs were then collected, anonymised, and copied.

The trial was approved by Bradford Research Ethics Committee (Reference number 10/H1302/60), and by Research and Development departments in the following Trusts and Health Boards: Betsi Cadwaladr University Health Board (no reference number); Blackpool, Fylde and Wyre Hospitals NHS Foundation Trust (RD0563); Cambridge University Hospitals NHS Foundation Trust (AO92132); Cardiff and Vale University Health Board (10/CMC/49); Cwm Taf Health Board (CT/118/10); East Lancashire Hospitals NHS Trust (2010/030); Lancashire Teaching Hospitals NHS Foundation Trust (1298); North Cumbria University Hospitals NHS Trust (124/10); University Hospitals of Morecambe Bay NHS Trust (SFRC 471). All participants provided written informed consent to take part in the study.

### Phase I: exploration of data

#### Procedure (I)

The clinical logs had been designed in line with generally accepted ‘best practice’ for bladder training and prompted voiding. Prior to starting the trial, a plan was developed for their analysis: the intention was to record the proportion of occasions on which the actual voiding time was within 30 min of the proposed voiding time. An initial exploratory phase of data input and analysis was undertaken in order to ascertain whether this method of fidelity assessment was feasible. One researcher (BC) undertook this exploratory phase of data input, piloting the proposed method and identifying issues, which were recorded and discussed by the authors.

#### Results (I)

Three major issues arose with the planned method of data analysis:

1. The voiding interval was sometimes either missing or documented as a range (e.g. “2 – 3 hourly”)
  Both of these did not constitute adherence to the protocol because the interval between voiding times was either not defined or not consistent. This meant that it was not possible to interpret whether or not the schedule of proposed voiding times was correct and consequently whether or not it had been followed.
2. Some proposed voiding times were missing
  Missing proposed voiding times prevented the research team from being able to assess whether an actual voiding time had occurred within 30 min of the corresponding proposed voiding time.
3. The intervals between proposed voiding times were incorrect
  Scheduled voiding intervals were sometimes miscalculated, for example a 3-hour voiding interval had been proposed yet voiding times were scheduled for 8 am, 10 am, 12 pm, etc. In these cases, assessing whether the actual voiding time had occurred within 30 min of the proposed voiding time was meaningless because the proposed voiding time was itself incorrect given the prescribed voiding interval.

The exploration of data suggested that, although documentation of an appropriate voiding interval and a correct schedule of proposed voiding times are prerequisites for successful undertaking of the SVP, these were often not done. Consequently, the original plan of assessing fidelity to treatment delivery through measuring the proportion of actual voiding times that had taken place within 30 min of their proposed voiding times was deemed no longer feasible. A new method was therefore developed, described in Phase II, in order to analyse the same fidelity data.

### Phase II: Revised fidelity assessment

#### Procedure (I)

Through exploring the reasons why the original method of fidelity assessment was not possible it became evident that certain ‘prerequisites’ had to be in place in order to perform the planned analysis. Working backwards, it was self-evident that a comparison of actual and proposed voiding times needed *correctly documented* proposed voiding times. In order to have a schedule of correctly documented proposed voiding times, it was necessary to have a *correctly documented* voiding interval. Having recognized these ‘prerequisites’, we used them to identify key quality indicators, which we believed would allow assessment of fidelity to treatment delivery, based upon completion of the clinical logs. Key quality indicators were assessed in stages, and are shown in Table 1 with their definitions. A filtering system was developed, whereby data input for an individual clinical log was terminated at one of two stages (Stage 1 or 2) if the key quality indicator for that stage had not been achieved. For each clinical log details of how it performed at each stage were recorded.

**Table 1:** Key quality indicators used to assess fidelity to treatment delivery as recorded on the clinical logs

Stage	Key quality indicator	Definitions of components
1.	VOIDING INTERVAL^a^:	The *voiding interval* is the specified length of time between each void for the patient for that day. The voiding interval determines the frequency of voiding throughout the day.
	Is the voiding interval present and appropriately documented?	*Appropriate documentation* refers to the documentation of an individual number (such as “2-hourly”) and not a range (such as “2 – 3 hourly”).
2.	PROPOSED VOIDING TIMES^a^:	*Proposed voiding times* should be documented at the start of each day, based upon the voiding interval. The proposed voiding times form a schedule of times for toileting, which healthcare staff should then try to follow.
	Are proposed voiding times present and documented correctly?	*Proposed voiding times ‘present’*: There should be no missing entries between the first and last documented proposed voiding time.
		*Proposed voiding times ‘documented correctly’*: Each interval between consecutive proposed voiding times should be identical to the voiding interval (e.g. 2 h between consecutive proposed voiding times for a voiding interval of “2-hourly”).
3.	ACTUAL VOIDING TIMES – within schedule:	The *actual voiding times* are the times at which the patient was toileted, and are recorded by healthcare staff.
	For how many proposed voiding times is the ‘actual voiding time’ documented and within 30 min?	The ‘gold standard’ for the ICONS intervention is that an *actual voiding time* should be within 30 min of a proposed voiding time.
4. (a)	GOOD PRACTICE – Encouragement: For how many proposed voiding times is the answer “YES” documented in response to the question “Did you give encouragement?”	For each voiding occasion, healthcare staff are required to indicate on the clinical log whether they have undertaken a number of ‘best practice’ components of the regime. These include giving encouragement to the patient and asking them whether they are wet (if on prompted voiding regime).
4. (b)	GOOD PRACTICE – Asking the patient if they are wet^b^:	
	For how many proposed voiding times is the answer “YES” documented in response to the question “Did you ask the patient if they were wet?”	

^a^A clinical log was not examined further if it did not achieve this stage

^b^This criterion refers to prompted voiding clinical logs only

For assessment of Stage 3 (the comparison of the actual voiding times to their corresponding proposed voiding times), any ‘clinically justifiable’ explanations documented were noted and adjustments made for them in the analysis. The main criteria for comments to be deemed ‘clinically justifiable’ were agreed between the authors and are shown in Additional file 1.

Data input was undertaken by one researcher (BC). The final analysis of the data was undertaken jointly by two researchers (BC and ML). A simple descriptive quantitative analysis was performed, exploring how well clinical logs from each trial arm had performed according to the key quality indicators.

#### Results (II)

A total of 396 clinical logs were analysed from Intervention sites and 320 from Supported Implementation sites, covering approximately 25 % of trial participants. The majority of clinical logs were for the prompted voiding programme. Results for fidelity to treatment delivery are shown in Table 2, categorised into Intervention and Supported Implementation trial arms (four sites in each arm).

**Table 2:** Results for fidelity to treatment delivery in the ICONS trial

Trial arm	Intervention^a^	Supported implementation
Number of clinical logs analysed	396	320
Number of patients	40	31
Percentage of total number of patients	24.4 % (40/164)	24.8 % (31/125)
Percentage of clinical logs according to type of regime: Prompted Voiding (PV)/Bladder Training (BT)	PV: 90.4 %	PV: 100.0 %
	/BT: 9.6 %	/BT: 0.0 %
STAGE 1: % with voiding interval present and correctly documented	83.3	89.4
STAGE 2: % with both voiding interval and schedule of proposed voiding times present and correctly documented	38.9	31.9
No. of clinical logs that achieved both Stage 1 and Stage 2	154	102
For the clinical logs that achieved both Stage 1 and Stage 2:		
STAGE 3: On average, how often was an ‘actual voiding time’ documented that was within 30 min of the proposed voiding time?^b^	54.8 %	56.0 %
STAGE 4a: On average, how often was encouragement documented as given?	58.4 %	57.5 %
STAGE 4b: On average, how often was it documented that the patient had been asked if they were wet?^c^	57.9 %	65.9 %

^a^This refers to sites which delivered the intervention only, and not the supported implementation package

^b^Occasions on which a clinically justifiable explanation was given for an early/late/missing actual voiding time were exempted from this analysis

^c^This applies to Prompted Voiding clinical logs only

The proportion of clinical logs that had both an appropriate voiding interval and a correct schedule of proposed voiding times documented was 38.9 % for the Intervention arm and 31.9 % for the Supported Implementation arm. This meant that 154 and 102 clinical logs passed both Stages 1 and 2 in the Intervention and Supported Implementation arms, respectively.

For clinical logs that passed Stages 1 and 2, the proportion of occasions on which an actual voiding time was documented that was within 30 min of the corresponding proposed voiding time was 54.8 % for the Intervention arm and 56.0 % for the Supported Implementation arm. For these clinical logs, which had passed Stages 1 and 2, it was documented that two key aspects of best practice, giving encouragement and asking the patient if they were wet, were performed on the majority of occasions.

**Fig. 1:**
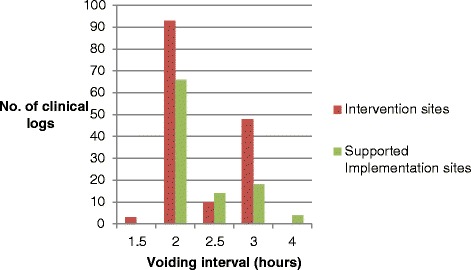
Bar chart to depict voiding intervals used by healthcare staff. The data in this Figure relate to clinical logs that achieved Stages 1 and 2 only

One principle of the SVP was that the voiding interval should be individualised and specific to each patient depending, for example, on their pattern of incontinence. The distribution of voiding interval frequencies for the clinical logs that passed Stages 1 and 2 (shown in Fig. 1) suggests that different voiding intervals were used, rather than a uniform interval being prescribed across all patients and days. The mean voiding interval was 2.33 h for the Intervention arm and 2.32 h for the Supported Implementation arm. The mean number of proposed voiding times documented each day was 5.08 and 4.98, respectively.

## Discussion

This paper describes the assessment of fidelity to treatment delivery of the ICONS systematic voiding programme (SVP). Assessment of fidelity to treatment delivery was based upon data collected from daily clinical logs completed by healthcare staff. Significant issues arose when the planned method of data analysis was trialled on the actual data collected: the voiding interval was often either missing or incorrectly documented and the schedule of proposed voiding times was either incomplete or incorrect. Both the voiding interval and schedule of proposed voiding times are prerequisites for undertaking the SVP. Therefore, the clinical logs had not been completed as intended. This meant that the *a priori* planned method for data analysis was unfeasible because it was often not possible to calculate the differences between actual and proposed voiding times. A new method was developed, in which key quality indicators were identified and an assessment made for each clinical log regarding its performance against each key quality indicator. This method appeared to work well and revealed overall a relatively low level of fidelity to treatment delivery.

The first objective of this study was to explore the feasibility of a method to assess fidelity to treatment delivery in the ICONS trial. The issues highlighted in the results from Phase I suggest that researchers should not assume that components of their intervention will necessarily be delivered in exactly the way they expect. In this trial, the voiding interval and schedule of proposed voiding times were not documented as expected. It is important for researchers to consider for every component of a complex intervention ways in which actual practice may deviate from the protocol, and take steps to minimise this. Researchers should also ensure that measurement of fidelity is sufficiently sensitive to capture meaningful variations to ‘core components’ of the intervention [23].

BCC recommendations state that interventions should be assessed against “*a priori* criteria” [1]. For the ICONS trial, *a priori* criteria were set, namely that actual voiding times should be within 30 min of their proposed voiding times. However, it was not foreseen that errors would arise in the documentation of both the voiding interval and the proposed voiding times, two prerequisites for both successfully undertaking the SVP and enabling the calculation of differences between actual and proposed voiding times. These issues rendered the planned method of fidelity assessment unfeasible. This demonstrates that, whilst it is important to develop plans for the assessment of fidelity to treatment delivery at the study design stage, it is necessary to remain flexible and adapt the planned method in light of unforeseen issues. Fidelity assessment should be an iterative process within trials [7].

The revised method of assessing fidelity to treatment delivery through the evaluation of clinical logs according to key quality indicators provided a feasible alternative. Despite the complexities of the information, data could be extracted from all clinical logs and used to assess fidelity to treatment delivery. However, this method of analysis has some drawbacks.

Clinical logs with a missing or incorrect voiding interval or an incomplete or incorrect schedule of proposed voiding times were not examined further. There were some clinical logs that contained only a small number of errors, for example just one or two incorrect proposed voiding times, but that otherwise demonstrated reasonably high levels of fidelity to treatment delivery, for example, by including a number of actual voiding times documented as within 30 min of their proposed voiding times. However, these data were not captured and consequently not included in the analysis. In a future trial, it would be useful to consider whether this method of data input could be amended to account for varying degrees of fidelity, rather than simply accepting or rejecting clinical logs at earlier stages of the process.

In this trial, *documentation* of delivery of the SVP was used as a proxy measure for fidelity to actual treatment delivery. Findings from other components of the process evaluation, for example interview data, suggest that healthcare staff did not always document everything that they did [17], and therefore it is likely that a method of fidelity assessment based upon healthcare staff recording of activity underestimates the true level of fidelity to treatment delivery. Similarly, it also appeared that at times healthcare staff found the amount of paperwork associated with the intervention onerous [17]; it is therefore worth considering whether the clinical logs could be simplified while still providing a structure for SVP delivery.

Some aspects of the clinical logs, such as documenting whether encouragement had been given, involved more subjective self-reporting by healthcare staff. There is therefore a risk of self-report bias in the use of clinical logs to measure fidelity, as self-report measures can often be inaccurate and may over-estimate fidelity [24–26]. Additionally, there is evidence from the clinical logs that selective documentation was sometimes undertaken, in which data were more likely to be missing if the required intervention component had not been achieved. For example, it appeared that sometimes healthcare staff would leave the answer to a question such as “Was encouragement given?” as missing rather than document “no”. In the future trial a more objective measure of fidelity may be appropriate, for example direct observation. However, this could affect intervention delivery as a consequence of providers being observed [26] and may not be feasible due to ethical issues inherent in observing patients receiving personal care.

A key limitation of the assessment of fidelity to treatment delivery in this trial was that data were not available regarding the total number of clinical logs per trial arm that should have been completed. It was not possible to calculate these numbers due to the complexities inherent in the delivery of the SVP. For example, sometimes patients would start and stop the SVP at various time points during their stay due to needing a catheter for a short period, or due to becoming temporarily medically unstable, and the dates for these variations were not consistently recorded. This meant that it was not possible to reliably calculate the theoretical numbers of clinical logs that should have been received. The first step in fidelity to delivery of the SVP was the completion of a clinical log and therefore a comparison of the numbers of clinical logs expected and received could add another stage to the assessment of fidelity to treatment delivery. In a future trial it will be important to develop a process that will ensure it is possible to calculate the number of clinical logs that should have been completed per sampling period, per trial arm.

A second objective of this study was to obtain preliminary evidence of fidelity to treatment delivery within the ICONS trial. Documentation of an appropriate voiding interval and a correct schedule of proposed voiding times (Stages 1 and 2) was often not done correctly. The original intention was to determine whether a patient had voided within 30 min of the proposed voiding time, but when this method proved unfeasible it was necessary to reconsider what constituted a reasonably valid method to assess ‘fidelity’. It was decided that the documentation of an appropriate voiding interval and a correct schedule of proposed voiding times were the first steps in the successful undertaking of the SVP and should therefore be the first steps in the assessment of fidelity to treatment delivery. The clinical logs that did not contain both these components (61.1 % in the Intervention sites; 68.1 % in the Supported Implementation sites) therefore lacked the necessary information for healthcare staff to be able to follow the SVP throughout the day and this constitutes a lack of fidelity. It is unclear why this happened, but one possible explanation is that healthcare staff misunderstood the principles of the SVP. In the future trial this needs to be addressed through detailed consideration of methods of implementation of the SVP, for example giving healthcare staff time to become familiar with and practice using the SVP before the trial intervention period begins [23, 27]. The future definitive ICONS trial will include a detailed plan for improved training provision, uptake and monitoring.

Whilst a key principle of the ICONS SVP is that voiding should occur within 30 min of a proposed voiding time, other aspects of treatment delivery were also important: healthcare staff should have given sufficient consideration to the most appropriate voiding interval for a particular patient on a particular day, and should also have attempted to undertake the SVP throughout the day, from 7.30 am until 9.30 pm. These components were only examined for clinical logs that achieved Stages 1 and 2. It was found that the length of the voiding interval did vary across days and across patients (data not presented), and this suggests that there was some individualisation of voiding interval. The mean number of proposed voiding times per clinical log was also relatively high for each arm, at around five for both arms, given that the voiding interval was almost always between 2-hourly and 3-hourly (as shown in Fig. 1) and that the SVP only ran from 7.30 am until 9.30 pm. This suggests that, on the whole, the SVP was undertaken through most of the day. Fidelity to these two components of the SVP therefore seems relatively high for clinical logs that passed Stages 1 and 2.

For clinical logs that achieved Stages 1 and 2, the percentage of occasions on which the actual voiding time occurred within 30 min of the proposed voiding time was around 55 %. A key principle of the SVP is that voiding occurs at a regular, consistent interval as part of a progressive voiding schedule [28, 29]; on a substantial proportion of occasions ICONS patients did not void at, or close to, the scheduled time. Fidelity to this principle of the SVP therefore appeared to be relatively low. One possible explanation for the relatively low fidelity is that returning to all patients on the SVP at their individualised times posed the most challenges to practice and was not always achievable. Additionally, there is evidence that staffing levels associated with weekend working can affect clinical outcomes [30], and it is possible that documentation of the SVP and fidelity to it could have been affected by different patterns of working/staffing at weekends versus in the week. However, exploring such patterns was beyond the scope of this paper. When fidelity to the intervention is low, the degree to which trial outcomes can be attributed to the intervention is compromised [31].

Fidelity to two ‘best practice’ components of the SVP, giving the patient encouragement and asking the patient if they were wet, was relatively high, although data for these components were extracted only from clinical logs that achieved Stages 1 and 2. Whilst these results are encouraging, they should be interpreted with caution as healthcare staff simply documented ‘yes’ or ‘no’ in relation to the two components, and, without asking for further detail to be provided about the manner in which these elements were provided, it is difficult to assess how accurate these measures are. There is also the risk of positive bias in self-reporting these more subjective elements of the programme [25].

A limitation of this study is that the analysis does not reflect the sampling strategy, which had two levels of clustering – by time and by patient. In our analysis we grouped together the clinical logs per site and then per trial arm and presented pooled results. The data were merged in this fashion in order to simplify the analysis and subsequent presentation. This method is unlikely to have affected our overall conclusions in relation to treatment fidelity, but would have implications if we were to perform any inferential statistics.

Key lessons have been learnt from the assessment of fidelity to treatment delivery in the ICONS trial. Real-life practice may often deviate from the protocol: the ICONS protocol for the SVP was often not followed and this highlights how important it is for researchers to consider how real-life practice may deviate from what was intended and explore ways in which to minimise this. An iterative approach to fidelity assessment is important in trials of complex interventions: ideally, fidelity data should be examined and analysed whilst the trial is ongoing, allowing issues to be addressed as the trial progresses. Different ways of defining and assessing fidelity should be considered: trials of complex interventions should consider the relative merits of subjective, self-reported measures of fidelity versus more objective measures, such as direct observation, and select the method most feasible and appropriate for the study. More comprehensive implementation strategies will be important in a future evaluation trial in order to improve fidelity, given that the overall fidelity to treatment delivery in the ICONS feasibility trial appeared to be relatively low.

## Conclusions

Fidelity to treatment delivery has been explored in detail in the ICONS feasibility trial. The feasibility of a proposed method for fidelity assessment has been investigated and lessons learnt from this experience will guide fidelity assessment in a future evaluation trial and may be of use to other research teams designing trials of complex interventions. Whilst it is important to agree upon strategies for implementing and assessing fidelity to treatment delivery during the design phase, researchers should still be prepared to adapt their planned methods during a trial in line with evolving issues. The assessment of fidelity should be an iterative process, with opportunities for fidelity data gathered to inform ongoing strategies to monitor and improve fidelity to treatment delivery. This exploration of fidelity to treatment delivery of the ICONS intervention (the SVP) has highlighted issues in the implementation of the SVP that will need to be addressed in a future trial. By addressing these issues it is possible that we will be able to improve healthcare staff fidelity to the protocol and potentially allow greater flexibility in delivery of the protocol amongst healthcare staff whilst not adversely impacting on the active components of the SVP.

## Additional file

Additional file 1:
**Criteria for comments to be deemed ‘clinically justifiable’.** This file provides details of the types of comments made by healthcare staff regarding early/late/missing voiding times that were or were not deemed ‘clinically justifiable’ by the research team. (DOCX 14 kb)

## Abbreviations

BCC: Behavior change consortium
BT: Bladder training
ICONS: Identifying continence options after stroke study
NIH: National Institutes of Health
PV: Prompted voiding
SVP: Systematic voiding programme
UC: Usual care
UI: Urinary incontinence
