# Supplementary material for: Assessing fidelity to treatment delivery in the ICONS (Identifying Continence OptioNs after Stroke) cluster randomised feasibility trial
Source: BMC Med Res Methodol. 2015 Aug 21;15:68. doi: 10.1186/s12874-015-0051-9 (PMC4546169; doi:10.1186/s12874-015-0051-9)
Supplement: Additional file 1: — Criteria for comments to be deemed ‘clinically justifiable’. This file provides details of the types of comments made by healthcare staff regarding early/late/missing voiding times that were or were not deemed ‘clinically justifiable’ by the research team. (DOCX 14 kb) [file 12874_2015_51_MOESM1_ESM.docx]

**Additional file 1 – Criteria for comments to be deemed ‘clinically justifiable’**

If there was not an actual voiding time documented that was within 30 minutes of its proposed voiding time, the research team recorded any ‘clinically justifiable’ comments that could potentially justify the lack of timely voiding. Below is the list of criteria for comments to be deemed ‘clinically justifiable’. A list of criteria for deeming comments as ‘not clinically justifiable’ is also provided.

**Types of comments that were deemed ‘clinically justifiable’**

- Patient sleeping
- Patient catheterised
- Patient not on the ward if location is*:
  - Out of hospital;
  - In hospital but NOT with an allied health professional;
  - Non-specified.

*In addition, the length of time off the ward should be either of sufficient length to preclude scheduled voiding or non-specified.

- Patient ‘refused’ or ‘did not want toilet’ *(underlying assumption associated with use of the words ‘refused’ and ‘want’ is that the patient was asked and therefore did not consent to voiding)*

**Types of comments that were NOT deemed ‘clinically justifiable’**

- Staffing issues / issues relating to being too busy
- Patient at physiotherapy or occupational therapy
- Patient lost buzzer
- Toileting time fell during meal time
